# Analysis of the acoustic environment in an ICU using patient information as a covariate

**DOI:** 10.1186/cc13206

**Published:** 2014-03-17

**Authors:** M Park, P Vos, A Kohlrausch, B Vlaskamp, AW Oldenbeuving

**Affiliations:** 1 Philips Research Laboratories, Eindhoven, the Netherlands; 2 St Elisabeth Hospital, Tilburg, the Netherlands

## Introduction

Noise levels in ICUs are often very high, potentially affecting patient health outcome, which are also considered to be among the risk factors contributing to ICU delirium [1]. In the current study, multivariate linear regression models were established to relate the acoustic data to the indicators of patient status.

## Methods

Acoustic measurements were taken in eight single-bed patient rooms in a level 3 ICU, while patient information was also collected, including APACHE IV score and mortality rate together with other potentially relevant variables; for example, the usage of mechanical ventilators, and so on. The hourly and daily trends of the acoustic data were obtained, and an analysis of variance was carried out to investigate the effect of relevant factors (for example, the time of day). Furthermore, multiple linear regression models were established to relate the different types of patient-related data (as independent variables) to each type of the acoustic data (as dependent variable), where independent variables were selected based on an exhaustive search method.

## Results

Data were collected for 3 months for 106 patients. The 24-hour trends of acoustic parameters corresponded well to the daily routine events in the ICU. The analyses for the first 4 days of the patients' ICU stay showed that the average SPL varied depending on the day (*P *= 0.023) and on the time of the day (day/night) (*P *< 0.001). The average noise level decreased from day 1 to day 2 with a significant reduction at night (*P *= 0.008), but it was found to rebound from day 2 where the increase of the daytime noise level from day 2 to day 4 was significant (*P *= 0.005). The results of the multiple linear regression showed that various patient conditions influenced different types of noise-level parameters. For example, the location of the patient room, the usage of mechanical ventilators and the mortality rate were found to correlate to the 10th-percentile SPL (L90) in the first 48 hours (adjusted *R*^2 ^= 0.58; *P *< 0.001). Also, the room location, gender and the usage of mechanical ventilators were found to be related to the 50th-percentile SPL (L50) in the same period (adjusted *R*^2 ^= 0.54; *P *< 0.001).

## Conclusion

Noise-level parameters were found to vary depending on the day of ICU stay, the time of day, and other indicators of the patient's status. For a rigorous analysis of the influence of noise on patients' outcome, the effects of these factors must first be controlled or corrected.
